# Supplementary material for: Prediction of urban heat island intensity based on multiple linear regression and deep learning
Source: PLoS One. 2025 Dec 29;20(12):e0339782. doi: 10.1371/journal.pone.0339782 (PMC12747439; doi:10.1371/journal.pone.0339782)
Supplement: Attachment — Submitted filename: Response to Reviewer 2 Comments.docx [file pone.0339782.s002.docx]

**For research article**

Response to Reviewer 2 Comments

Dear Reviewers,

Thank you very much for your review of our manuscript and your valuable comments. We have carefully read every comment you have made and have revised and added it accordingly. The following is the reply and modification instructions for each of your comments:

**Point-by-point response to Comments and Suggestions for Authors**

**Comments 1: Integrating CA-Markov and artificial neural networks for UHII-related predictions is not new. The authors should better clarify the limitations of previous studies or explain why those approaches may be less suitable for the current context. More importantly, they need to highlight their model's distinct contributions. This should be discussed more clearly in both the introduction and discussion sections.**

**Response 1: We appreciate your insightful comments on the novelty of combining CA-Markov and artificial neural networks for UHII prediction. While this approach isn't entirely new, our study innovatively integrates it with spectral indices and the MLR model to predict future UHII. We'll highlight our model's unique contributions in the introduction and discussion.**

**Comments 2: Although the authors provide R² and RMSE to evaluate the models, it is recommended to include additional indexes such as MAE and MAPE to better assess performance. Furthermore, the training process lacks information regarding model generalization and overfitting control. Including training and validation loss curves to demonstrate model convergence and stability.**

**Response 2: We thank you for your suggestions on model evaluation. We'll consider adding MAE and MAPE in future research. Regarding model generalization and overfitting control, we'll strive to provide more detailed information.**

**Comments 3: In the multiple linear regression section, while the relationship between indices like NDVI and UHII is described, the statistical robustness of the regression is not fully demonstrated. The manuscript should report the p-values for regression coefficients and VIF values to test for significance and multicollinearity.**

**Response 3: We appreciate your advice on reporting p-values and VIF values. We'll consider incorporating these statistical measures in future studies to strengthen the robustness of our regression analysis.**

**Comments 4: For the Landsat image processing, the authors must specify which spectral bands were used and clarify whether cloud masking and atmospheric correction were applied before further analysis. Regarding the UHII definition, it is essential to explain how the boundaries between urban and suburban areas were determined. Additionally, the manuscript should consistently use Celsius or Kelvin for temperature.**

**Response 4: We thank you for your attention to the details of Landsat image processing. We'll clarify in future studies which spectral bands were used and whether cloud masking and atmospheric correction were performed. We'll also unify the temperature units.**

**Comments 5:A confusion matrix or Kappa coefficient can provide a more accurate classification performance assessment. Since subsequent prediction results rely heavily on land use classification, its uncertainty and potential impact should be discussed.**

**Response 5: We acknowledge your suggestion about using confusion matrices or Kappa coefficients. We'll consider providing a more detailed discussion of classification uncertainty and its potential impact on subsequent predictions.**

**Comments 6:In the spectral index computation (NDVI, NDBI, NDWI, BSI), the authors mention using the Google Earth Engine (GEE) but do not clarify the temporal range (e.g., annual mean, summer average, specific dates). The figures showing index time series (e.g., Fig. 4) should indicate the temporal resolution and include units.**

**Response 6: We appreciate your comments on the calculation of spectral indices. We'll specify the time range used for calculating the indices and ensure that future figures include time resolution and units.**

**Comments 7:The meaning of “time step t=3” should be clarified—does this refer to three consecutive periods? If so, is a sliding window input approach used?**

**Response 7: We thank you for highlighting the need to clarify the meaning of "time step t=3." We'll provide a clearer explanation in our future work.**

**Comments 8:There is redundancy between Table 3 and Figure 15, as both display similar information. One of them may be removed or merged for conciseness.**

**Response 8: We appreciate your suggestion to remove or merge redundant tables and figures. We'll strive to maintain clarity and conciseness in our presentation of results.**

**Comments 9:A table listing the complete CNN-LSTM-Attention model hyperparameters, such as batch size, learning rate, number of epochs, and optimizer settings.**

**Response 9: We thank you for suggesting the inclusion of a table listing the hyperparameters of the CNN-LSTM-Attention model. We'll consider providing this information in future studies.**

**Comments 10:Please add a glossary of technical terms.**

**Response 10: We appreciate your suggestion to include a glossary of technical terms. We'll consider adding this to improve the accessibility of our research.**

Thank you again for your review and valuable comments on our study. If you have any further comments or suggestions, please feel free to make them.

Yours sincerely,
