# Supplementary material for: Prediction of urban heat island intensity based on multiple linear regression and deep learning
Source: PLoS One. 2025 Dec 29;20(12):e0339782. doi: 10.1371/journal.pone.0339782 (PMC12747439; doi:10.1371/journal.pone.0339782)
Supplement: Attachment — Submitted filename: Response to Reviewer 3 Comments.docx [file pone.0339782.s003.docx]

**For research article**

Response to Reviewer 3 Comments

Dear Reviewers,

Thank you very much for your review of our manuscript and your valuable comments. We have carefully read every comment you have made and have revised and added it accordingly. The following is the reply and modification instructions for each of your comments:

**Point-by-point response to Comments and Suggestions for Authors**

**Comments 1:** Taking for granted the above, I only a minor comment: in your introduction, together with "Green Wedge Urbanism", and "Low-Carbon Urbanism", you could find interesting the "Isobenefit Urbanism" (IU) approach too regarding the UHI due to its constant ratio between green and built land regardless the settlement size (from village to megacity).

**Response 1:** Thank you for this valuable suggestion. We agree that Isobenefit Urbanism (IU) offers a meaningful perspective. However, given the journal's strict length constraints for the Introduction section, we find it challenging to adequately elaborate on IU's theoretical dimensions without making this part overly fragmented. We have briefly touched upon the equity issues of cross-scale planning in the Discussion section, and we hope to dedicate a future paper to exploring how IU can be integrated with our research methodology in greater depth.

Thank you again for your review and valuable comments on our study. If you have any further comments or suggestions, please feel free to make them.

Yours sincerely,
